# Ground-Active Arthropod Diversity Under Energycane and Biomass Sorghum Production

**DOI:** 10.3390/insects16050442

**Published:** 2025-04-23

**Authors:** Yubin Yang, Tanumoy Bera, Hamid Araji, Fugen Dou, Lloyd T. Wilson, William L. Rooney, Jesse I. Morrison, Brian S. Baldwin, Joseph E. Knoll, John L. Jifon, Alan L. Wright, Dennis C. Odero, Hardev S. Sandhu, Anna L. Hale, Himaya P. Mula-Michel, Jing Wang

**Affiliations:** 1 Texas A&M AgriLife Research and Extension Center, 1509 Aggie Drive, Beaumont, TX 77713, USA; 2 Department of Soil and Crop Sciences, Texas A&M University, College Station, TX 77843, USA; 3 Department of Plant and Soil Sciences, Mississippi State University, Starkville, MS 39762, USA; 4 Crop Genetics and Breeding Research Unit, United States Department of Agriculture-Agricultural Research Service, 115 Coastal Way, Tifton, GA 31793, USA; 5 Texas A&M AgriLife Research and Extension Center, 2415 E Hwy 83, Weslaco, TX 78596, USA; 6 Indian River Research and Education Center, University of Florida, 2199 South Rock Road, Fort Pierce, FL 34945, USA; 7 Everglades Research and Education Center, University of Florida, 3200 East Palm Beach Road, Belle Glade, FL 33430, USA; 8 Sugarcane Research Unit, United States Department of Agriculture-Agricultural Research Service, 5883 USDA Road, Houma, LA 70360, USA

**Keywords:** energycane, biomass sorghum, arthropod richness, Shannon diversity, Simpson diversity, environmental covariates

## Abstract

Energycane and biomass sorghum are two of the most promising cellulosic energy crops in the southeastern US. Research on these two energy crops has focused mainly on biomass production, and there is a lack of knowledge on their ability to promote biodiversity and ecosystem services. This paper presents results from a comprehensive study on ground-active arthropod diversity in seven sites across five states in the southeastern US (Florida, Georgia, Louisiana, Mississippi, and Texas). Pitfall traps were deployed four times during each crop season for energycane, biomass sorghum, and a local reference conventional crop from 2020 to 2022. Arthropod abundance (individuals/(trap × day)) values were 4.9 ± 0.46, 3.7 ± 0.18, and 2.6 ± 0.16 (mean ± stderr) for conventional crops, biomass sorghum, and energycane, respectively, with a significant difference found only between conventional crops and energycane. Individuals were identified to arthropod orders, and Hill’s diversity indices were calculated based on the number of individuals in each arthropod order instead of the number of individuals in each arthropod species. Order-based arthropod richness values were 5.3, 5.2, and 4.8 for biomass sorghum, conventional crops, and energycane, with significant difference found only between biomass sorghum and energycane. There was no significant difference in the order-based Shannon diversity and Simpson diversity between the three crop types. The effective number of arthropod orders for the two energy crops decreased from 5.0 to 3.4 to 2.9 with increasing order of diversity from arthropod richness to Shannon diversity to Simpson diversity. The explained variability by environmental factors also decreased with increasing Hill’s order of diversity. The results from this study indicate no significant advantage in order-based arthropod diversity in growing biomass sorghum and energycane. This research fills a critical knowledge gap in understanding the impacts of cellulosic energy crop production on biodiversity and ecosystem services.

## Simple Summary

Energycane and biomass sorghum are two of the most promising cellulosic energy crops in the southeastern US. Research on both crops has focused mainly on biomass production, and there is a lack of information on their ability to promote biodiversity and ecosystem services (i.e., direct and indirect benefits to human society). This paper presents results from a comprehensive study on arthropod diversity in seven sites across five states in the southeastern US (Florida, Georgia, Louisiana, Mississippi, and Texas). Traps were deployed four times to capture ground-dwelling arthropods in energycane, biomass sorghum, and a local reference conventional crop. Daily catches on the number of individuals per trap were 4.9, 3.7, and 2.6 for conventional crops, biomass sorghum, and energycane, respectively. Order-based arthropod richness values were 5.3, 5.2, and 4.8 for biomass sorghum, conventional crops, and energycane, respectively. The results from this study indicate a lack of a significant difference in order-based arthropod diversity between these crops.

## 1. Introduction

Biofuel production in the United States has historically focused on grain-based first-generation feedstocks and is mostly concentrated in the Midwestern States [1]. Second-generation biofuels are derived from cellulosic energy crops such as dedicated energy crops and agriculture and forestry residues [2]. According to the 2023 Billion-Ton Report [3], the United States has the potential to produce one billion dry tons of biomass annually from agricultural, forestry, waste, and algal materials. Biomass-based fuels could displace approximately 30% of the 2005 US petroleum consumption without negatively impacting the production of food and other agricultural products [3]. Biomass from dedicated energy crops could account for approximately 50% of the potential growth by 2030 [4]. The most promising cellulosic energy crops include switchgrass (*Panicum virgatum* L.), giant miscanthus (*Miscanthus × giganteus* (Greef & Deuter ex Hodkinson & Renvoize)), energycane (*Saccharum* spp. interspecific hybrids), biomass sorghum (*Sorghum bicolor* (L.) Moench) [5], and short-rotation woody crops [4]. Switchgrass is a native warm-season perennial grass that can grow over a wide geographical range [6,7]. Giant miscanthus is a long-living perennial grass with cold tolerance [8]. Once planted, miscanthus can remain in the ground for fifteen to twenty years [9].

Energycanes are interspecific hybrids derived from crosses between *Saccharum officinarum* L. (sugarcane) and *S. spontaneum* L. (wild sugarcane). They tend to have greater cold tolerance, higher fiber, and lower sugar content compared to commercial sugarcane [10,11]. They are well suited for production in the southeast US, especially in the high-rainfall, low-lying heavy-clay soils on which most crops cannot grow well [11,12]. As a perennial crop similar to sugarcane, they can produce a plant cane and 4–6 productive ratoon crops [13].

Biomass sorghums are photoperiod-sensitive hybrids that have excellent drought tolerance and high water-use efficiency [14]. As an annual crop similar to grain sorghum, photoperiod-sensitive biomass sorghum genotypes have a long growing season [14,15]. Most studies on these energy crops have focused on biomass production potentials [16,17,18], economics [19,20,21], carbon sequestration [22], and life cycle assessment [23,24]. Studies examining ecosystem impacts such as changes in faunal and floral biodiversity in feedstock production systems have focused primarily on switchgrass and miscanthus [25,26,27,28,29,30].

Several biodiversity studies have been conducted in miscanthus production systems in Europe. Semere and Slater [31] reported that the positive effects of miscanthus and reed canary-grass (*Phalaris arundinacea*) on invertebrates in England were mainly through the indirect use of weeds as food resources and habitats. Radzikowski et al. [32] found that arthropod diversity was lower in cup plant (*Silphium perfoliatum* L.) compared to fallow land, miscanthus, and woody energy crops. In a study on carabid assemblages in cup plant, Virginia mallow (*Sida hermaphrodita* L.), and tall wheatgrass (*Thinopyrum ponticum* Podp.) in Bavaria, Germany, Burmeister [33] found no significant differences in arthropod abundance or ground beetle species richness between perennials and annuals. A follow-up paper reported increased arthropod abundance and diversity after the harvest of these perennial crops [34].

Biodiversity studies in the US have been mostly focused on switchgrass. Gardiner et al. [35] reported that bees were three to four times more abundant in switchgrass and prairie than in maize, and coccinellids were generally most abundant in prairie and switchgrass; the authors concluded that plant-diverse bioenergy cropping systems can support a higher abundance and diversity of beneficial insects. Werling et al. [36] found that switchgrass and prairie fields harbored significantly more plant species, methanotrophic bacteria, arthropods, and birds than maize fields. Helms et al. [37] found that native perennial biofuel crops supported up to 185% more ant species than maize fields and provided up to 55% more natural pest suppression. Haan et al. [38] found that biodiversity gains over maize in plants and most animal groups were larger in mixed than in single perennial systems; the authors also found that species richness in two energy sorghum-based systems tended to be similar to or lower than maize.

Except for a study on earthworm diversity in sugarcane in Brazil [39] and on biodiversity involving high-sugar energy sorghum in the Midwestern US [38], there has been no research reported on the biodiversity under cellulosic biomass sorghum and energycane production systems. We hypothesize that arthropod abundance and biodiversity in energycane should be similar to that in sugarcane and that abundance and biodiversity in biomass sorghum should be similar to that in grain sorghum under similar production practices. This paper presents the results of a comprehensive study on the diversity of ground-active arthropods in energycane, biomass sorghum, and conventional crop production systems in seven sites across five states in the southeastern United States (Florida, Georgia, Louisiana, Mississippi, and Texas). This study is the first multi-state assessment of ground-active arthropods in energycane and biomass sorghum systems. It fills an important knowledge gap regarding the biodiversity impacts of second-generation biofuel crops in the southeastern US.

## 2. Materials and Methods

### 2.1. Site Descriptions

Field studies were conducted in Texas (Beaumont, College Station, and Weslaco), Mississippi (Starkville), Georgia (Tifton), Florida (Belle Glade), and Louisiana (Houma) (Table 1 and Supplementary Figure S1). Except for Houma, which grew only energycane, each of the sites grew both energycane and biomass sorghum. Beaumont is characterized by high-clay soil and high precipitation (1398 mm/year). College Station is characterized by clay loam soil with approximately 853 mm of annual precipitation. Weslaco has sandy clay loam soil and a subtropical climate normally with frost-free winters and low annual precipitation of 684 mm/year. Starkville has sandy loam soil, with a 1123 mm average annual precipitation, with regular freezing periods in winter. Tifton is characterized by loamy sand soil, with approximately 1030 mm of annual precipitation. Belle Glade has Histosols with more than 60% organic carbon and a 1217 mm average annual precipitation. Houma receives an average of 1598 mm of annual precipitation and has variable soil conditions ranging from loam to high-clay soils with a very shallow water table. These sites are representative of the range of soil, weather, and production conditions across the southeastern US.

### 2.2. Energycane and Biomass Sorghum Genotypes

Six of the sites grew energycane genotypes Ho 01-08, Ho 02-113, and Ho 06-9002 each year from 2021 to 2023, all of which were developed by the USDA-ARS Sugarcane Research Unit in Houma, Louisiana [40]. Due to quarantine restriction delaying the movement of energycane from Louisiana, Bell Glade grew Ho 02-113, UFCP 84-1047, and UFCP 87-0053, with the last two jointly developed by the University of Florida and USDA-ARS Sugarcane Field Station at Canal Point in Florida [41,42].

Five photoperiod-sensitive hybrid genotypes of biomass sorghum developed by the Sorghum Breeding Program at Texas A&M AgriLife Research (College Station, TX, USA) were used in this study. TAM17501, TAM17651, and TAM08010 were grown in 2020, and TAM08001, TAM08005, and TAM08010 were grown from 2021 to 2023. When planted after the spring equinox, their vegetative growth continues for about 180 days until the fall equinox when panicle initiation is triggered by daylengths less than 12.5 h, allowing for a longer period of biomass accumulation [43].

### 2.3. Experimental Design

A randomized complete block design was used for each energy crop in each site. Two of the seven sites (Beaumont and Starkville) conducted experiments with two nitrogen (N) rates, consisting of a low rate and a site-specific recommended rate. Each of the two sites had 48 plots (2 energy crops × 3 genotypes × 2 N × 4 replicates). The remaining five sites (Belle Glade, College Station, Houma, Tifton, and Weslaco) had a single site-specific recommended N rate. Each of the five sites had 24 plots (2 energy crops × 3 genotypes × 1 N × 4 replicates), except for Houma, which grew only energycane and had 12 plots. Each site also included a conventional reference crop with four replicates for comparison with the two energy crops on biodiversity and environmental sustainability. Conventional crops included rice (*Oryza sativa*) in Beaumont, sugarcane in Belle Glade and Houma, maize (*Zea mays*) in Starkville and Tifton, and grain sorghum in College Station and Weslaco. Insecticides were applied to biomass sorghum and conventional crops, except for sugarcane and energycane (Supplementary Table S1). Herbicides were applied for weed control for all sites and crops (Supplementary Table S1). No irrigation water was applied, except for Beaumont with furrow irrigation and Weslaco with drip irrigation. Nitrogen application was based on local recommend rates and varied among sites (Table S1). Weather data for each site during the experiment were collected from a nearby weather station.

### 2.4. Pitfall Trap Design and Ground-Active Arthropod Sample Collection

A double-layered design was used for the pitfall traps [44]. The outer layer consisted of a PVC pipe that was 152 mm in both height and internal diameter. The upper rim of the PVC pipe was lined with 6 mm diameter Teflon tubing that was split along its length to slide onto the PVC pipe’s upper rim. The PVC pipe was placed inside a hole dug into the bed of an interior crop row. The internal layer consisted of a transparent plastic funnel and a transparent plastic collection jar. The plastic funnel had a top opening of 152 mm and a bottom opening of 50 mm. The funnel top rested on the Teflon tubing. The collection jar had an internal diameter of 114 mm and a height of 102 mm with a capacity of 700 mL. Each collection jar was placed inside the PVC pipe and was half-filled with 50/50 antifreeze (ethylene glycol). A transparent plastic plate (254 mm diameter), supported by wooden dowels, was placed above the pitfall trap to serve as a rain shelter.

Ground-active arthropods were collected using pitfall traps from two replicate plots for each energy or conventional crop treatment. Pitfall traps were deployed, and samples were collected four times per year from 2020 to 2022, once at planting or at the start of the crop season, twice during the crop season, and once immediately before crop harvest. A total of 839 traps were collected during the 2020–2022 crop seasons, with 278, 444, and 117 traps for energycane, biomass sorghum, and conventional crops, respectively (Table 2). Beaumont and Starkville had the largest number of traps due to an additional low-nitrogen treatment. Houma had the smallest number of traps due to not growing biomass sorghum. Average durations for each trap deployment were 15, 15, and 14 days for energycane, biomass sorghum, and conventional crops, respectively (Table 2).

For each deployment, one pitfall trap was placed on an internal row, at least 15 m away from the field edge. The same placement location was used for all four deployments. For each deployment, pre-labelled collection jars were half-filled with 50/50 antifreeze and placed under the funnels inside the PVC tubes and retrieved two weeks later. The retrieved collection jars were tightly capped and placed in leakproof plastic bags and shipped to Beaumont. Pitfall trap samples were stored in a cool storage room (10 °C) before processing and specimen identification.

### 2.5. Pitfall Trap Sample Processing

A pitfall trap sample was poured onto a sieve. The sample on the sieve was rinsed with tap water to clean any dirt or mud and then rinsed back into the original container with 70% ethanol solution. The sample in the ethanol solution was poured onto a filter paper placed inside a Buchner funnel to facilitate quick drainage of the solution. Arthropod samples collected in the funnel were transferred and spread over one or more filter papers, using tweezers when needed. A Sony DSC-RX100 20 MP digital camera (Tokyo, Japan) was used to photograph each filter paper along with a sample ID. An in-house C# program was then used to add 24 square line grids to each photo. IrfanView (https://www.irfanview.com/, accessed on 20 April 2025) was used to open each photo, and arthropods in each square grid were counted by experienced staff with identification to taxonomic orders [45].

### 2.6. Data Analysis

Daily trap catch was calculated as the total number of individuals divided by the trap deployment duration. Abundance-based pairwise dissimilarity between the three crop types was calculated using the R package *betapart* [46]. Pairwise dissimilarity is a measure of how two assemblages differ from each other in terms of species composition [47,48].

We used *Hill Numbers* as a measurement of biodiversity [49,50]

(1) HillNumbers: Dq≡∑i=1Spiq1/(1−q)

where *D* is the diversity measure, $p_{i}$ is the proportional abundance of species i, *S* is the number of species, and *q* is the order of diversity (q=0, 1, 2). The Hill number provides a unified framework for measuring biodiversity [49,50,51]. A Hill diversity of order 0 represents species richness, which represents the number of species in an assemblage. A Hill diversity of order 1 is the Shannon diversity, which weights individuals equally. A Hill diversity of order 2 is the Simpson diversity, which places greater weights on dominant species. These measures are referred to as the effective number of species [50,52], which tends to decrease with increasing order of diversity, since a higher order of diversity places greater emphasis on the most dominant species. The diversity profile as a function of diversity order reflects the characteristics of a specific assemblage [52]. Since each index examines diversity from a different perspective, we presented results for all three orders of diversity so that we could have a more comprehensive picture of how they differed among the different crop production systems.

Due to the large number of specimens collected, we were only able to identify individuals to arthropod orders. Adopting the approach in [53], we calculated Hill’s biodiversity measures and pairwise dissimilarity based on the number of individuals in each arthropod order instead of the number of individuals in each species. Throughout the manuscript, the results on the arthropod richness, Shannon diversity, Simpson diversity, and pairwise dissimilarity were based on arthropod orders instead of species.

Analyses of the daily trap catch and diversity (arthropod richness, Shannon diversity, and Simpson diversity) were conducted using SAS GLM (v. 13.2) [54]. Square root transformation was applied to the trap catch [33]. The main factors were site, year, nitrogen level, crop type, and season. Crop type includes energycane, biomass sorghum, and a conventional crop as a reference crop. Season was categorized into early season (samples collected before 31 June) and late season (samples collected after 31 June). Treatment means were compared with Tukey’s multiple comparison test in SAS with α = 0.05 [54].

Analysis of variance indicated significant effects of site, year, and/or season on arthropod abundance and Hill’s orders of diversity. To determine the main environment covariates associated with site, year, and season, we calculated four sets of environmental covariates, including the following: (1) soil; (2) weather factors during the trap deployment duration; (3) weather factors during the 3 months before trap retrieval; and (4) weather factors during the crop year. Soil factors included soil texture (sand, clay, and silt %), soil organic matter, and soil pH. Weather factors included average temperature, average relative humidity, average precipitation, and average solar radiation. Collinearity between the environment covariates was diagnosed using the COLLIN and VIF options in SAS REG [54]. Covariates with a Condition Index exceeding 30 and a VIF value exceeding 10 were excluded [55,56]. Thereafter, stepwise regression in SAS GLMSELECT [54] was used to identify the most significant covariates impacting the daily catch and species diversity [32]. The covariates that were included in the stepwise regression included Clay, SoilOM, AvgRainTrap (average rainfall during the trap duration), AvgTempSeason (average temperature during the 3 months prior to trap retrieval), AvgRainSeason (average temperature during the 3 months prior to trap retrieval), and AvgRainYear (average precipitationduring the crop year).

## 3. Results

### 3.1. Arthropod Abundance Across Sites, Years, and Crop Types

A total of 43,218 ground-active invertebrates were caught over the three crop seasons, of which 43,024 were identified as belonging to arthropods, 116 were identified as non-arthropods, and 78 were not identified due to specimen decay or camera angle. This analysis focused solely on arthropods (Table 2). Of the 43,024 arthropods, 10,377, 24,771, and 7876 were caught in energycane, biomass sorghum and conventional crops, respectively (Table 2).

**Figure 1:**
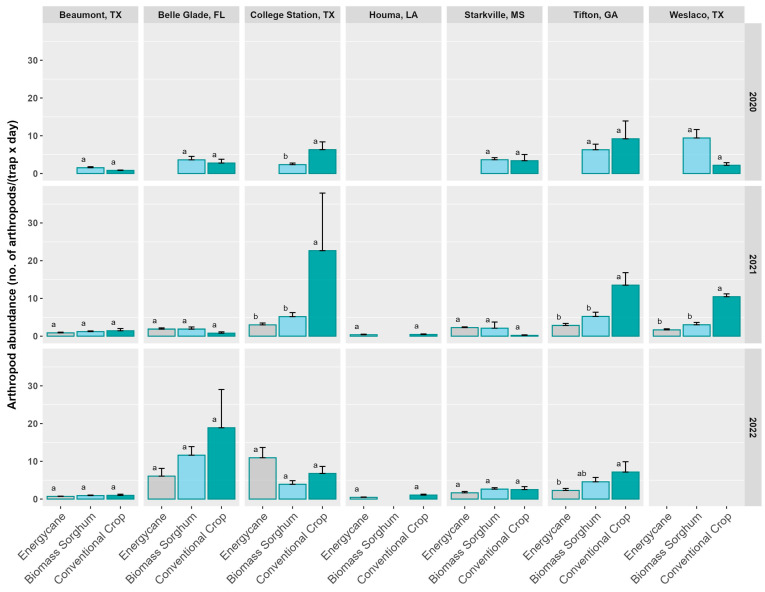
Arthropod abundance (no. arthropods/(trap × day)) from pitfall traps across seven sites and in three years (2020–2022) for three crop types. Crop types include energycane, biomass sorghum, and conventional crops. Conventional crops included rice for Beaumont, sugarcane for Belle Glade and Houma, maize for Starkville and Tifton, grain sorghum for College Station, and cotton (2020) and grain sorghum (2021–2022) for Weslaco. Crop types at a site and year having the same lowercase letter were not significantly different from each other at 0.05 with Tukey’s HSD multiple comparison test. Error bars in the figure represent standard errors. Different color bars represent different crop types for easy visual differentiation.

In 2020, the lowest arthropod abundance (individuals/(trap × day)) was observed in the conventional crop in Beaumont; the highest abundance was observed in biomass sorghum in Weslaco, followed by the conventional crop (maize) in Tifton (Figure 1). Except for College Station, which had significantly higher arthropod abundance in the conventional crop than in biomass sorghum, there was no significant difference in arthropod abundance between biomass sorghum and the conventional crop for all other sites.

In 2021, the lowest abundance was observed in the conventional crop in Starkville; the three highest arthropod abundance were observed in conventional crops in the order of College Station (grain sorghum), Tifton (maize), and Weslaco (grain sorghum) (Figure 1). College Station, Tifton, and Weslaco all had significantly higher arthropod abundances in conventional crops than in either energycane or biomass sorghum; no significant differences were observed among crop types for the other site and crop type combinations (Figure 1).

In 2022, the lowest abundance was observed in energycane in Houma; the highest abundance was observed in the conventional crop (sugarcane) in Belle Glade, followed by energycane in College Station and biomass sorghum in Belle Glade (Figure 1). Tifton had a significantly higher arthropod abundance in the conventional crop than in energycane; no significant differences were observed among crop types for the other site and crop type combinations.

### 3.2. Arthropod Distribution Across Taxonomic Orders

**Figure 2:**
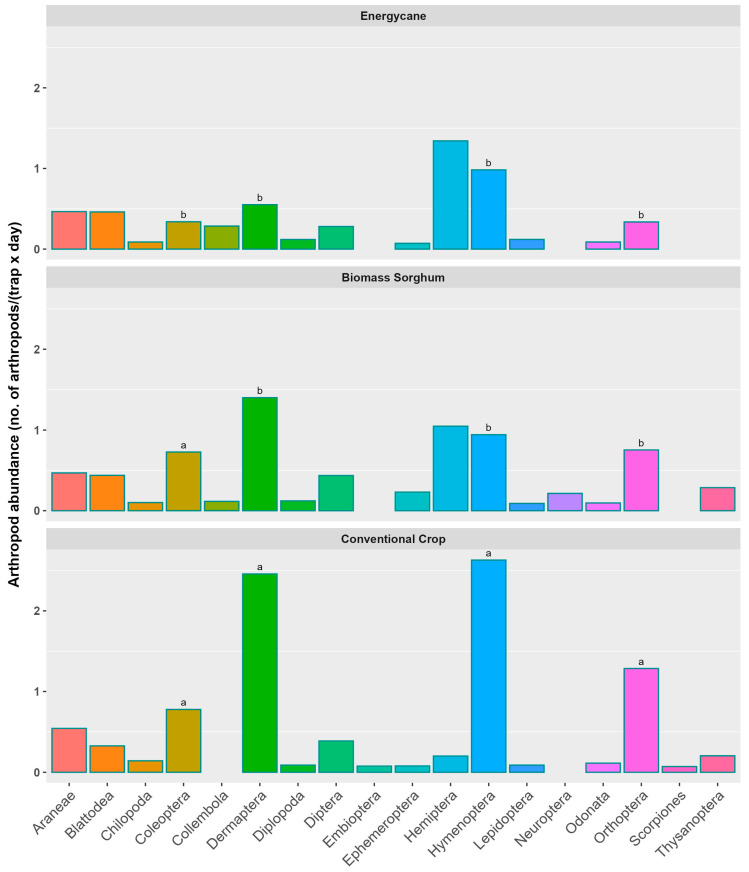
Distribution of arthropods among different taxonomic orders across three crop types (data pooled across sites and years). Arthropod abundance among crop types for a specific taxonomic order in a vertical column having the same lowercase letter were not significantly different from each other at 0.05 with Tukey’s HSD multiple comparison test; letters are omitted for taxonomic orders with no significant difference in arthropod abundance among crops. Different color bars represent different arthropod orders for easy visual differentiation.

In energycane, Hemiptera had the greatest abundance, followed by Hymenoptera, Dermaptera, and Araneae (Figure 2). In biomass sorghum, Dermaptera had the greatest abundance, followed by Hemiptera, Hymenoptera, and Orthoptera (Figure 2). In conventional crops, Hymenoptera had the greatest abundance, followed by Dermaptera, Orthoptera, and Coleoptera (Figure 2). Coleoptera abundance in conventional crops and biomass sorghum was significantly higher than in energycane. Dermaptera abundance in conventional crops was significantly higher than in energycane and biomass sorghum. Both Hymenoptera and Orthoptera abundance in conventional crops was significantly higher than in energycane and biomass sorghum (Figure 2). Although there were considerable differences in the abundance of Hemiptera among the three crop types, no significant differences were detected (Figure 2).

A separate analysis on arthropod abundance for individual crops revealed that Coleoptera abundance in corn was significantly higher than in energycane, cotton, and rice, and Orthoptera abundance in grain sorghum was significantly higher than in the other six crops (Supplementary Figure S2). Other than that, there were no significant differences in abundance for any of the other arthropod orders among the different crops. Although there were considerable differences in the abundance of Hymenoptera and Dermaptera among the different crops, no significant differences were detected (Supplementary Figure S2).

### 3.3. Abundance-Based Pairwise Dissimilarity

**Figure 3:**
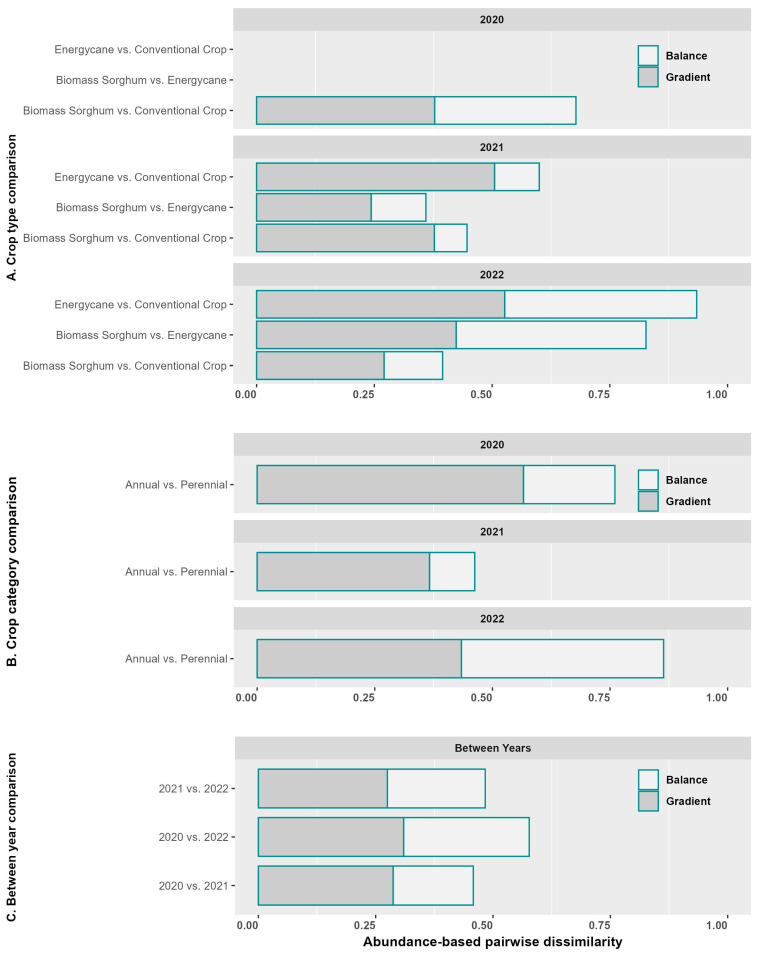
Abundance-based pairwise dissimilarity. (**A**) Crop type comparison (no energycane in 2020); (**B**) crop category comparison; (**C**) between-year comparison.

We examined abundance-based pairwise dissimilarity between crop types (energycane, biomass sorghum, and conventional crops) by pooling the communities across sites (Figure 3A). The greatest dissimilarity was found between energycane and conventional crops in 2022, followed by biomass sorghum vs. energycane in 2022 and biomass sorghum vs. conventional crops in 2020. The least dissimilarity was found between biomass sorghum vs. energycane in 2021, followed by biomass sorghum vs. conventional crops in 2022. There was considerable variability in dissimilarity between the years. Dissimilarity for energycane vs conventional crops or biomass sorghum in 2022 was much greater than in 2021 (Figure 3A). It was not clear whether this represents a temporal trend due to the perennial nature of energycane or just random variation between years.

The dissimilarity measure can be partitioned into two components, including balanced variation in abundance and abundance gradient. The abundance-based *balance* and *gradient* components are analogous to the replacement and nestedness-resultant components of incidence-based dissimilarity [46,47,48,57,58]. For biomass sorghum vs. conventional crops, the gradient and balance components were very similar in 2020, but the gradient component dominated the dissimilarity in 2021 and to a lesser extent in 2022 (Figure 3A). For energycane vs conventional crops, the increased dissimilarity from 2021 to 2022 was dominated by an increase in the balance component. For biomass sorghum vs energycane, the increased dissimilarity in 2022 was due to the increase in both the balance and the gradient component, with greater increase being contributed by the balance component (Figure 3A).

We also examined pairwise dissimilarity between annual and perennial crops by pooling data across crop categories and sites (Figure 3B). The greatest dissimilarity was found in 2022, followed by 2020, with 2021 having the smallest dissimilarity (Figure 3B). In 2020 and 2021, the gradient component for annual vs. perennial was ~3 times the balance component, but the gradient and balance components were very similar in 2022 (Figure 3B). The pairwise dissimilarity of 2020 vs. 2022 was larger than those of 2020 vs. 2022 and 2021 vs. 2022 (Figure 3C). The balance and gradient components were approximately the same between years. The pairwise dissimilarity between years was smaller than those between crop types or crop categories (Figure 3).

### 3.4. Impact of Sites, Years, and Crop Types on Arthropod Abundance

The main effect factors of site, year, and crop type had a significant impact on arthropod abundance, accounting for 21.4, 1.6, and 0.7% of the total variability, respectively (Table 3). Nitrogen level and season were not significant. The most significant two-way interactions were site × year, site × crop type, and site × season, accounting for 8.1, 4.6, and 2.0% of the total variability, respectively. The main effects and two-way interactions accounted for 40.3% of the total variability (Table 3). All main effects, two-way interactions, and three-way interactions accounted for a total of 46.7% of the total variability. The inclusion of additional multi-way interactions did not substantially increase the explained variability.

### 3.5. Order-Based Arthropod Richness, Shannon’s Diversity, and Simpson’s Diversity

**Figure 4:**
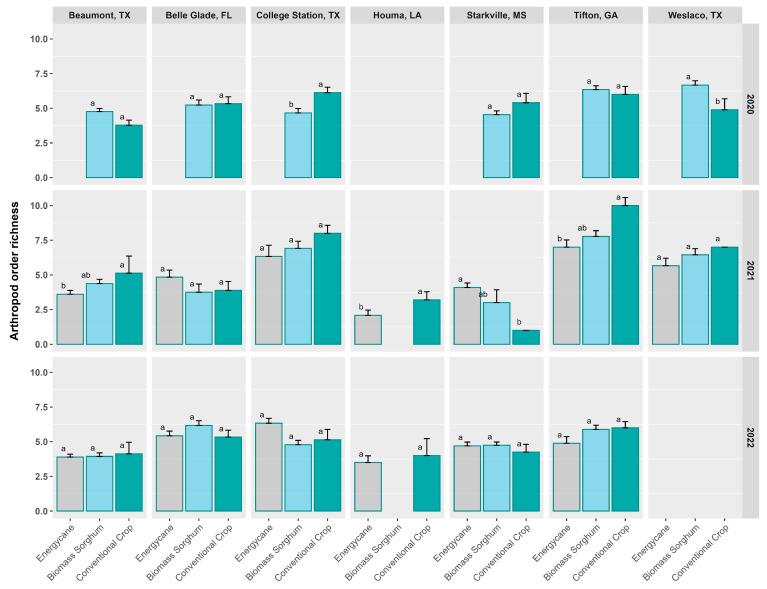
Order-based arthropod richness from pitfall traps across seven sites and in three years (2020–2022) for three crop types. Crop types at a site and year having the same lowercase letter were not significantly different from each other at 0.05 with Tukey’s HSD multiple comparison test. Error bars in the figure represent standard errors. Different color bars represent different crop types for easy visual differentiation.

In 2020, arthropod richness ranged from 6.8 (Weslaco) to 4.8 (College Station) for biomass sorghum and from 6.4 (College Station) to 3.9 (Beaumont) for conventional crops (Figure 4). Arthropod richness for conventional crops (grain sorghum) was significantly greater than for biomass sorghum in College Station, but the reverse was true for Weslaco. For all other sites, there was no significant difference in arthropod richness between the two crop types.

In 2021, arthropod richness ranged from 7.4 (Tifton) to 1.9 (Houma) for energycane, from 7.9 (Tifton) to 2.8 (Starkville) for biomass sorghum, and from 10.0 (Tifton) to 1.5 (Starkville) for conventional crops (Figure 4). Arthropod richness for conventional crops was significantly greater than for energycane in Beaumont, Houma, and Tifton, but the reverse was true for Starkville. Across all sites, there was no significant difference in arthropod richness between biomass sorghum and conventional crops. In 2022, there was no significant difference in arthropod richness between the three crop types (Figure 4).

Arthropod richness only considers the number of categories in an assemblage (i.e., the number of taxonomic orders in our case) and ignores abundance. Shannon diversity assigns equal weight to each individual, while Simpson diversity assigns greater weight to dominant species. Averaged over the three years and across the seven sites, the Shannon diversities were 3.6, 3.4, and 3.3 for biomass sorghum, energycane, and conventional crops, respectively, with no significant differences between the three crop types (Supplementary Figure S3); the Simpson diversities were 3.0, 2.9, and 2.8 for biomass sorghum, energycane, and conventional crop, respectively, also with no significant differences (Supplementary Figure S4).

### 3.6. Impact of Sites, Years, and Crop Types on Order-Based Arthropod Diversity

The main effects of site and season had a significant impact on arthropod richness, accounting for 21.0 and 0.6% of the total variability (Table 3). Year, crop type, and nitrogen level were not significant. Site × year, site × season, and site × crop type were the top three most significant two-way interactions, accounting for 7.0, 6.6, and 2.5 of the total variability, respectively. The main effects and two-way interactions accounted for a total of 41.2% of the total variability (Table 3). All main effect factors and two- and three-way interactions account for a total of 47.1% of the total variability. The inclusion of additional multi-way interactions did not substantially increase the explained variability.

**Figure 5:**
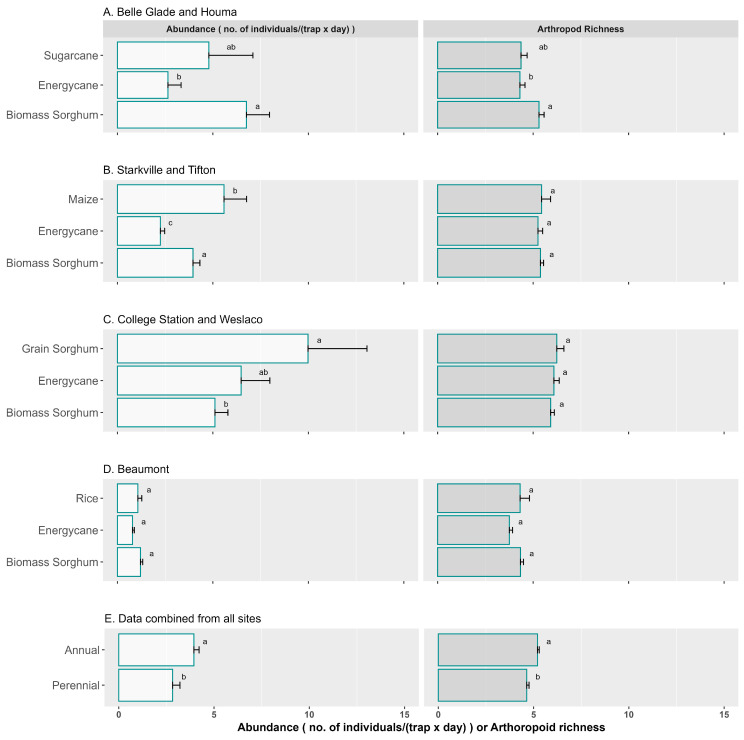
Abundance and order-based arthropod richness for energy and conventional crops: panels (**A**–**D**): using data from sites that shared the same conventional crop; panel (**E**): using data for annuals or perennials from all sites. Crops in each plot having the same lowercase letter were not significantly different from each other at 0.05 with Tukey’s HSD multiple comparison test. Error bars in the figure represent standard errors.

An additional analysis was conducted using data from sites that shared the same conventional crop (Figure 5A–D). Based on data from Belle Glade and Houma (Figure 5A), the daily trap catch for biomass sorghum was significantly higher than for energycane, but no difference was observed between energycane and sugarcane nor between sugarcane and biomass sorghum. Based on data from Starkville and Tifton (Figure 5B), the daily catch was highest for maize, followed by biomass sorghum and energycane, with significant differences among all three crops. Based on data from College Station and Weslaco (Figure 5C), grain sorghum had the highest daily trap catch, followed by energycane, and biomass sorghum, with grain sorghum being significantly higher than biomass sorghum. Based on data from Beaumont, the only site that had rice as a reference crop (Figure 5D), there was no significant difference among rice, energycane, and biomass sorghum. For sites that shared the same conventional crop, there was no significant difference in arthropod richness, Shannon diversity, and Simpson diversity between conventional crops and energy crops except for the Belle Glade and Houma data set (Figure 5A), which showed significantly higher arthropod richness for biomass sorghum than energycane but not sugarcane.

When energycane, biomass sorghum, and conventional crops were grouped between annuals and perennials and pooled across all sites and years, the mean arthropod abundance values were 3.9 and 2.8 for annual and perennial, respectively (Figure 5E). The mean arthropod richness was 5.3 for annual and 4.7 for perennial. Annual crops had significantly greater arthropod abundance and richness than perennial crops (Figure 5E).

### 3.7. Impact of Environmental Factors on Arthropod Abundance and Order-Based Arthropod Diversity

**Figure 6:**
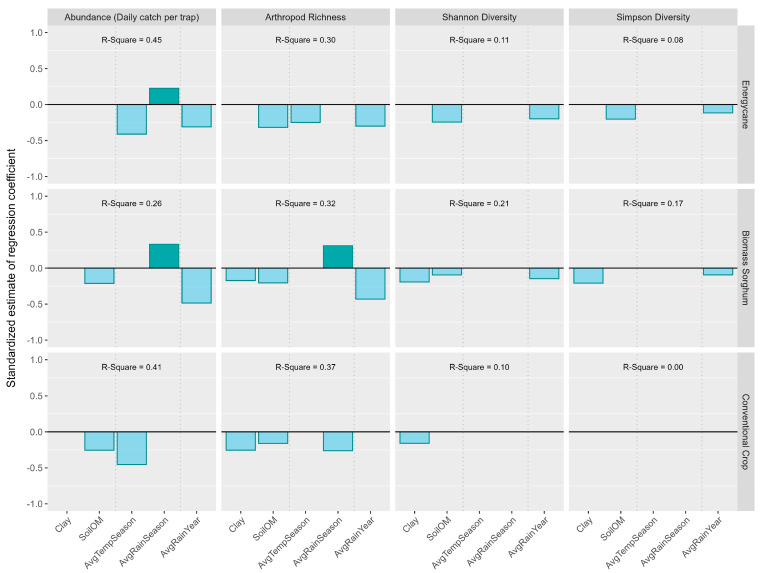
Impacts of environmental covariates on arthropod abundance and order-based arthropod diversity (bar plots showing significant environmental covariates through stepwise regression). Darker color bars represent positive effect and lighter color bars represent negative effect.

Average rainfall during the trap season was positively correlated with arthropod abundance and arthropod richness for energycane and biomass sorghum but negatively correlated with arthropod richness for conventional crops (Figure 6). Soil clay content, soil organic matter, average temperature during the trap season, and average rainfall during the crop year were all negatively correlated with arthropod abundance and biodiversity regardless of crop types (Figure 6). With an increasing order of diversity from arthropod richness to Shannon diversity to Simpson diversity, there was a decreasing trend in the explained variability for energycane (*R*^2^: 0.30 → 0.11 → 0.08), biomass sorghum (*R*^2^: 0.32 → 0.21 → 0.17), and conventional crops (*R*^2^: 0.37 → 0.10 → 0.00) (Figure 6). There was also a decreasing trend in the number of environmental factors that had a significantly positive or negative correlation with the diversity index for energycane (3 → 2 → 2), biomass sorghum (4→ 3 → 2), and conventional crops (3 → 1 → 0).

**Figure 7:**
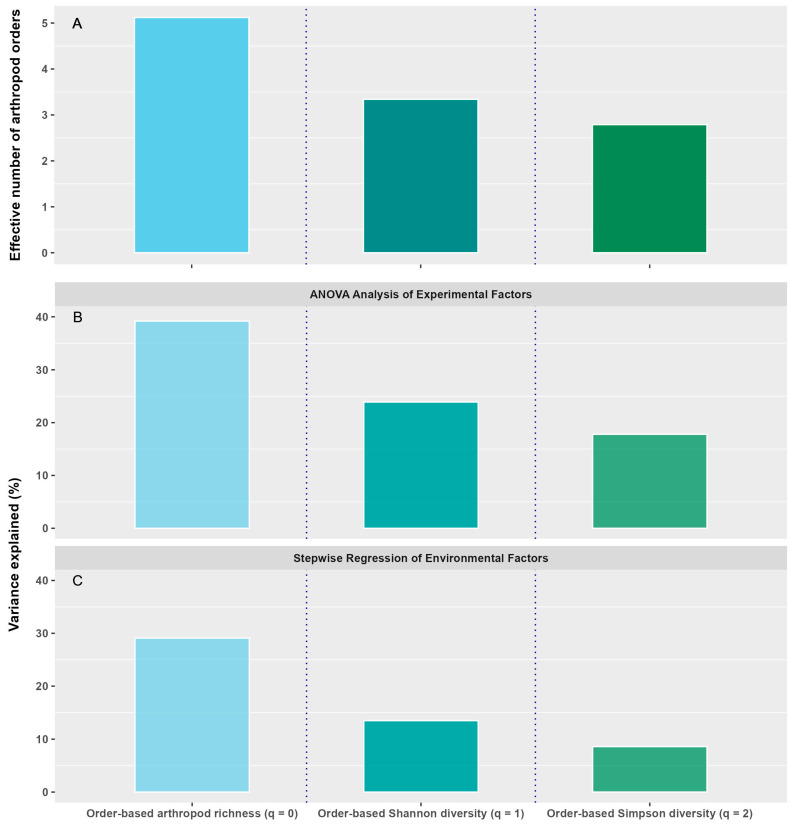
Hill’s order of diversity in relation to effective number of arthropod orders (**A**) and variance that can be explained through ANOVA of experimental factors (**B**) and stepwise regression of environmental covariates (**C**) (data pooled across sites, years, and crop types).

The effective number of taxa decreased from 5.0 to 3.4 to 2.9 with an increasing order of diversity from arthropod richness to Shannon diversity to Simpson diversity (Figure 7A). There was also a decreasing trend in the explained variability with the increasing order of diversity. The main effects and two-way interactions from the analysis of variance accounted for 39.2, 23.9, and 17.8% of the total variability for arthropod richness, Shannon diversity, and Simpson diversity, respectively (Figure 7B). Stepwise regression with environment covariates explained 29.1, 13.5, and 8.6% of the total variability for arthropod richness, Shannon diversity, and Simpson diversity, respectively (Figure 7C), which is much less than the amount of variability explained with the ANOVAs. This suggests that the environmental covariates did not completely capture the variability due to site, year, and season. The stepwise regression also showed a decreasing explained variability with increasing order of diversity (Figure 7C) and a decreasing number of environmental factors that had a significantly positive or negative correlation with the diversity measure (Figure 6). These results suggest less sensitivity to environmental factors with increasing emphasis on dominant taxa [33].

## 4. Discussion

### 4.1. Cellulosic Energy Crops and Biodiversity

First-generation biofuel crops refer to those that contain starch, sugar, or oil, whereas second-generation crops include lignocellulosic crops and fast-growing trees [2]. In a global synthesis analysis, Tudge, Purvis, and De Palma [27] found that compared to sites with primary vegetation, species richness and abundance were 37% and 49% lower for first-generation biofuel crops and 19% and 25% lower for second-generation biofuel crops. Similar results were also reported in a global metanalysis by Núñez-Regueiro, Siddiqui, and Fletcher [28].

Higher biodiversity in second-generation crops has been mostly linked to the complexity in their vegetation structures. Platen et al. [59] found that species richness and biodiversity were greatest in perennial energy crops and lowest in silage maize; the authors attributed the positive impacts of energy crops on biodiversity to the increased complexity of the vegetation structure. Invertebrates are the basic components of agricultural biodiversity and are often associated with weed vegetation [60]. High plant species richness is associated with more diverse and more complex arthropod communities [61]. Semere and Slater [31] found that field margins and open biomass crop fields tend to harbor a greater number of invertebrate families due to a more diverse plant complex. Similar results were also reported in a meta-analysis by Batáry et al. [62]; the authors found that field margins and hedgerows are more than twice as effective in promoting biodiversity as in-field measures such as organic management. Dauber et al. [63] showed that patchiness of miscanthus fields resulted in higher weed cover and thus higher biodiversity promotion.

In this study, we assessed the arthropod diversity of two cellulosic crops (energycane and biomass sorghum), in comparison with conventional crops, using data from three years across seven sites in the southeastern United States. Compared to conventional crops, we found no major advantage in arthropod biodiversity growing biomass sorghum and a slightly negative effect from growing energycane. Arthropod abundance in 2021 was significantly higher in conventional crops than in either energycane or biomass sorghum in College Station, Tifton, and Weslaco (Figure 1), but there were no significant differences in arthropod order richness for these sites (Figure 4). This was probably due to a dominance in abundance of Dermaptera, Hymenoptera, and Orthoptera in conventional crops (Supplementary Figure S2).

This study involved seven experiment sites in five southeastern US states. Due to the unique production environment at each site, we were only able to have common reference crops among some sites. Since reference crops may differ in their vegetation structures, they may consequently attract different ground-dwelling arthropods, potentially introducing bias to the results. To examine this potential bias, we conducted separate analysis using data from sites that shared the same conventional crop (Figure 5); we reached a similar conclusion, indicating no significant advantage in growing bioenergy crops, suggesting the potential bias may be minimal.

Both energycane and biomass sorghum fields were managed following conventional crop production practices with effective weed and pest control. Stand establishment for both was excellent, with no obvious patchiness for voluntary weed growth. Field margins were also well maintained, with little weed establishment. The surrounding areas were either grown with conventional crops or fallow lands. The intensive conventional management style and the mostly monoculture surrounding landscape might have contributed to the observed lack of biodiversity gain for both energycane and biomass sorghum in this study [25,64,65]. Designing energy crop production systems that foster local and landscape heterogeneity may promote biodiversity and agricultural sustainability [63,64,66,67]. Werling et al. [36] found that the linkage between biodiversity and ecosystem services depends not only on the choice of bioenergy crops but also on the local landscape context and surrounding habitats. The authors advocated for a bioenergy policy that supports multiuse diversified agricultural landscapes and promotes multiple critical ecosystem services. This concept was further promoted through a comprehensive review by Englund et al. [68] on the benefits of multifunctional perennial production systems for bioenergy production.

### 4.2. Biodiversity in Perennial vs. Annual Crops

Perennial crops usually require less inputs than annual crops, especially for crops cultivated in long production cycles [32,69]. The reduced input requirements include pesticides, fertilizers, water, and soil disturbance through tillage or cultivation, which can potentially provide greater habitat stability and increased diversity and abundance of arthropods [34,36,68,70,71].

Biodiversity data from recent studies comparing perennials and annuals seem to provide mixed results. Werling et al. [36] found significantly more plant species, methanotrophic bacteria, arthropods, and birds in switchgrass and prairie fields than in maize fields; they concluded that perennial grasslands enhance biodiversity and multiple ecosystem services in bioenergy landscapes. Haan, Benucci, Fiser, Bonito, and Landis [38] found that for plants and most animal groups, biodiversity gains over maize were distinctly larger in mixed than in single perennial systems. Burmeister [33] found that the abundance and species richness of ground beetles in perennials compared to annuals were site-specific, and there were no pronounced differences in species compositions and biodiversity. Radzikowski, Matyka, and Berbec [32] reported that arthropod diversity differed across years, and there was no clear evidence that growing perennial energy crops affects arthropod diversity. Williams and Feest [9] reported that spider and ground beetle diversity in miscanthus was lower compared to adjacent conventional agricultural ecosystems. In a meta-analysis involving three perennial grasses (miscanthus, switchgrass, and reed canary grass), Lask, Magenau, Ferrarini, Kiesel, Wagner, and Lewandowski [25] reported positive but non-significant trends in arthropod and bird diversity.

The difference in conclusions from these studies could be attributed to the types of perennials and annuals involved and the surrounding landscape. The perennial crops in this study included energycane and sugarcane. The annual crops included biomass sorghum, maize, cotton, grain sorghum, and rice. The perennial crops had significantly less arthropod abundance and arthropod richness and a lower Shannon diversity, but there was no significant difference in Simpson diversity between the annuals and perennials (Figure 5E). Biomass sorghum (Figure 5A), maize (Figure 5B), and grain sorghum (Figure 5C) all had high arthropod abundance, which might have contributed to the significantly greater measures for the annual crops. There was no difference in Shannon diversity and Simpson diversity between the crops. Except for rice, annual crops, including maize, grain sorghum, and biomass sorghum, have relatively tender leaves, which may provide a better food source for arthropods and may possibly contribute to the greater arthropod abundance and diversity.

### 4.3. Taxonomic Sufficiency and Recognized Taxonomic Units in Biodiversity Analysis

Due to the large number of specimens collected in this study, we were only able to identify individuals to arthropod orders. Arthropod richness, Shannon diversity, and Simpson diversity were calculated based on the number of individuals in each arthropod order. Order-level resolution could potentially obscure meaningful diversity patterns at the family, genius, or species level [72,73,74] and could be partially attributed to the relatively weak differences between energy and conventional crops or between annual vs. perennial crops observed in this study. Calculating Hill numbers using order-level data could introduce a taxonomic resolution bias, as they typically underestimate species richness (the q=0 Hill number) because multiple species are aggregated into a higher taxon. Additionally, measures that account for relative abundances (with q>0) may appear more even than they really are since the variation among species within orders is obscured. As a result, the diversity measures are “smoothed out” by losing the finer-scale heterogeneity present at the species level.

Several studies have addressed the taxonomic sufficiency [72,73,74] and used the concepts of morphospecies or recognized taxonomic units in biodiversity analysis to address the challenge in identifying specimens to species [72,73,75,76,77,78]. A morphospecies refers to a group of organisms that are classified as a species based primarily on their morphological (physical) characteristics [75,79]. A recognizable taxonomic unit is a more flexible, broader concept. It refers to any unit of classification (species, subspecies, population, or even a morphospecies) that can be distinguished from others based on morphology, genetics, or other distinguishing features [80]. In a study to determine whether morphospecies or recognizable taxonomic units can provide reasonable estimates of biodiversity in comparison with formal Labin binomials, Olive and Beattie [75] found that the error using morphospecies was 13% for spiders and 6% for ants in species identification. Derraik et al. [76] reported that the correlation between morphospecies and species was 91% for Lepidoptera but only 63% for Coleoptera and 50% for Araneae. In a study on arthropod diversity, Biaggini et al. [78] reported that the use of order-level classification could clearly distinguish among main land use types based on their faunal composition and diversity. Furthermore, order-level analyses gave outcomes comparable to those obtained with using Carabidae species. Conversely, analyses with family-level data for Coleoptera did not reveal any distinction among land types [78]. The authors concluded that order surrogacy could be seen as a preliminary approach in assessing arthropod diversity in agricultural landscapes. A further study would be to explore how different recognizable taxonomic units or ranks in the hierarchical classification system impact arthropod biodiversity measures in energycane and biomass sorghum production systems.

### 4.4. Ground- and Vegetation-Dwelling Taxa

This study used pitfall traps to assess the abundance and diversity of ground-active arthropods. A meta-analysis by Marja, Tscharntke, and Batary [67] indicated that diversified agri-environments (organic farming, wildflower strips/areas, and grassy field margins) support both arthropod abundance and species richness. However, the observed pattern was primarily driven by vegetation-dwelling (mainly bees, bumblebees, or butterflies) and not soil-dwelling (mainly carabids, beetles, or spiders) arthropod taxa, which are less mobile compared to vegetation-dwelling taxa.

In a long-term study to assess the effects of grassland management and manipulation on biodiversity, Weisser et al. [81] reported that the diversity of most organisms responded positively to increases in plant species richness, and the effect was stronger for aboveground than for belowground organisms. In another long-term study spanning 25 years, Jonason et al. [82] found that butterfly abundance increased gradually over time, approaching a 100% increase. In contrast, the vegetation-dwelling arthropods did not show a clear temporal effect. Hüber et al. [83] found that compared to maize monoculture, intercropping maize–bean resulted in significant increases in bee activity and species richness, but carabid arthropod abundance and species richness did not differ significantly between the cropping systems. Our study only examined ground-dwelling arthropod taxa under energycane and biomass sorghum production systems. A further study could include both vegetation-dwelling and ground-dwelling arthropods. In addition, our study included energycane production through the second year (first ratoon). Longer-term experiments might provide greater insights into temporal dynamics of ground-dwelling arthropod abundance and diversity in energycane production systems.

Most studies on abundance and biodiversity have focused on the correlation between invertebrate abundance and diversity with flora diversity [9,31,33,35,60,63,81] and agronomic practices [32]. There have been limited quantitative studies on how environmental covariates impact arthropod abundance and diversity [84,85]. By accounting for the timing of peak abundance for carabid beetle species, Honek [85] found a significant effect of temperature on carabid activity; the carabid catch size increased by an average of 6.3% per 1 °C increase in average temperature. Our study and related works [84,85] highlight the importance of and need for incorporating environment covariates in studying abundance and species diversity.

## 5. Conclusions

This paper presents the results of a comprehensive study on ground-active arthropod diversity across seven sites in the southeastern United States, involving three contrasting production systems, including energycane, biomass sorghum, and conventional crops. Arthropod abundance was significantly higher in conventional crops than in energycane, but there was no significant difference in abundance between biomass sorghum and conventional crops. Order-based arthropod richness in biomass sorghum was significantly higher than in energycane, but there was no significant difference in arthropod richness between biomass sorghum and conventional crops. Our results suggest no major advantage in order-based arthropod richness or biodiversity growing biomass sorghum and a slightly negative effect of growing energycane. With the increasing order of Hill’s diversity, we observed a decreasing difference in diversity measures and a decreasing explained variability, suggesting less sensitivity to environmental factors with increasing emphasis on dominant orders. The results from this study indicate no significant advantage in order-based arthropod biodiversity growing biomass sorghum and energycane. This research fills a critical knowledge gap in understanding the impacts of cellulosic energy crop production on biodiversity and ecosystem services.

## Figures and Tables

**Table 1:** Site information and average weather conditions (2020–2022).

Site	State	Lat (°N)	Long (°W)	Elevation (m)	Annual Precipitation (mm)	Daily Average		
						Temperature (°C)	Humidity (%)	Radiation (MJ/m^2^)
**Weslaco**	Texas	26.2	97.9	20	684	23.8	65.8	16.2
**Belle Glade**	Florida	26.7	80.6	3	1217	23.7	68.2	16.2
**Houma**	Louisiana	29.6	90.8	1	1598	21.1	67.8	16.0
**Beaumont**	Texas	30.1	94.3	10	1398	21.0	79.1	16.1
**College Station**	Texas	30.5	96.4	67	853	21.5	65.3	16.5
**Tifton**	Georgia	31.5	83.6	107	1030	20.0	67.7	14.7
**Starkville**	Mississippi	33.4	88.7	79	1123	18.0	67.5	15.4

**Table 2:** Total trap deployments and total catches of ground-active arthropods (summary over 2020–2022).

Location	Total Number of Trap Deployments				Total Number of Captured Arthropods			
	Energycane *	Biomass Sorghum *	Conventional Crop *	Total Traps	Energycane	Biomass Sorghum	Conventional Crop	Total
**Beaumont, TX, USA**	91 × 14	136 × 14	26 × 13	253	1015	2290	358	3663
**Belle Glade, FL, USA**	46 × 15	54 × 16	23 × 16	123	2539	5248	2024	9811
**College Station, TX, USA**	30 × 14	71 × 14	20 × 14	121	3961	3752	2642	10,355
**Houma, LA, USA**	26 × 15		9 × 15	35	147		100	247
**Starkville, MS, USA**	32 × 16	82 × 15	15 × 15	129	887	3561	555	5003
**Tifton, GA, USA**	35 × 17	53 × 16	12 × 14	100	1405	4116	1584	7105
**Weslaco, TX, USA**	18 × 14	48 × 15	12 × 16	78	423	5804	613	6840
**Total**	278 × 15	444 × 15	117 × 14	839	10,377	24,771	7876	43,024

* Number of traps × trap duration in days.

**Table 3:** Analysis of variance and percentage of variance explained by the main effects and two-way interactions on arthropod abundance (no. individuals (trap × day)) and order-based arthropod diversity.

Main Effects and Two-Way Interactions	Arthropod Abundance			Order-Based Arthropod Diversity		
	DF	(Square Root Transformed)		DF	Arthropod Richness	
		*p*	Variance (%)		*p*	Variance (%)
Site	6	<0.001	21.4	6	<0.001	21.0
Year	2	<0.001	1.6	2	0.680	0.1
Crop Type	2	0.009	0.7	2	0.344	0.2
Nitrogen Level	1	0.514	0.0	1	0.080	0.2
Season	1	0.530	0.0	1	0.004	0.6
Site × Year	10	<0.001	8.1	10	<0.001	7.0
Site × Crop Type	11	<0.001	4.6	11	<0.001	2.5
Site × Nitrogen Level	1	0.967	0.0	1	0.309	0.1
Site × Season	5	<0.001	2.0	5	<0.001	6.6
Year × Crop Type	3	0.192	0.4	3	0.414	0.2
Year × Nitrogen Level	2	0.169	0.3	2	0.020	0.6
Year × Season	2	0.002	1.0	2	0.001	1.3
Crop × Nitrogen Level	1	0.298	0.1	1	0.586	0.0
Crop × Season	2	0.565	0.1	2	0.005	0.8
Nitrogen Level × Season	1	0.979	0.0	1	0.463	0.0
Total (Explained)			40.3			41.2
Model DF	50			50		
Error DF	775			775		

## Data Availability

The original contributions presented in this study are included in the article/Supplementary Materials. Further inquiries can be directed to the corresponding author (contact author: Yubin Yang; yyang@aesrg.tamu.edu).

## Supplementary Materials

The following supporting information can be downloaded at: https://www.mdpi.com/article/10.3390/insects16050442/s1, Table S1: Crop management^1^ and pesticide applications. Figure S1: Experiment sites and crops. Figure S2: Distribution of arthropods among different taxonomic orders across different crops in three years (2020–2022). Figure S3: Order-based Shannon diversity from pitfall traps across seven sites and in three years (2020–2022) for three crop types. Figure S4: Order-based Simpson diversity from pitfall traps across seven sites and in three years (2020–2022) for three crop types.
