# Supplementary material for: Ground-Active Arthropod Diversity Under Energycane and Biomass Sorghum Production
Source: Insects. 2025 Apr 23;16(5):442. doi: 10.3390/insects16050442 (PMC12112514; doi:10.3390/insects16050442)
Supplement: Supplementary file 1 [file insects-16-00442-s001.zip › insects-3539026-supplementary.pdf]

Table S1. Crop management[1] and pesticide applications.

Site	Crops	Insecticides	Herbicides	Irrigation	Nitrogen (Kg/ha)
**Beaumont**	Energycane	-	Metribuzin, 2-4-D	Furrow	56, 168
	Biomass sorghum	Sivanto	Dual, Atrazine	Furrow	56, 168
	Rice	Mustang Maxx	Command, Permit	Flood	207
**Belle Glade**	Energycane	-	None	None	0
	Biomass sorghum	Thimet 20G, Warrior, Coragen	Atrazine, Dual	None	73
	Sugarcane	-	Atrazine, Armezon	None	0
**College Station[2]**	Biomass sorghum	Sivanto	Dual, Huskie	None	168
	Grain sorghum	Sivanto	Dual, Huskie	None	168
**Houma**	Energycane	-	Metribuzin, Prowl	None	120
	Sugarcane	-	None	None	120
**Starkville**	Energycane	-	Prowl, Atrazine, 2-4-D	None	50, 200
	Biomass sorghum	Mustang Maxx	Dual, Atrazine, Prowl	None	50, 200
	Corn	-	Charger Max, Atrazine, Broadloom, Prowl	None	200
**Tifton**	Energycane	-	Atrazine, 2-4 D, Prowl	None	50
	Biomass sorghum	Sivanto	Dual, Atrazine, Basagran	None	160
	Corn	Counter 20G	Charger Max, Atrazine, Cornerstone, Basagran	Overhead	250
**Weslaco**	Energycane	-	Metribuzin, Valor, Prowl	Drip	168
	Biomass sorghum	Prevathon	Atrazine, Prefar, Prowl	Drip	168
	Grain sorghum	Transform WG	Prefar	Drip	168

[1] All sites used conventional tillage; [2] No data on energycane due to pool stand establishment.

Supplementary Figures

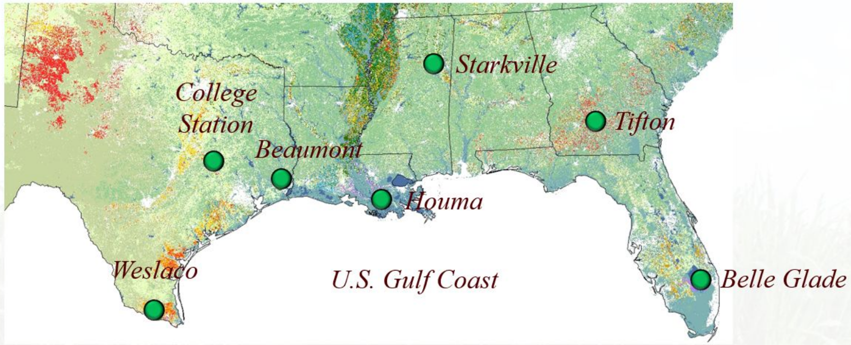

Location	*Energycane*	*Biomass Sorghum*	*Conventional Crop*
Weslaco, TX	*Cultivars (3)*	*Cultivars (3)*	*Grain Sorghum*
College Station, TX	*same*	*same*	*Grain Sorghum*
Beaumont, TX	*same*	*same*	*Rice*
Houma, LA	*same*	-	*Sugarcane*
Starkville, MS	*same*	*same*	*Corn*
Tifton, GA	*same*	*same*	*Corn*
Belle Glade, FL*	*same**	*same*	*Sugarcane*

**Florida's phytosanitation laws required planting existing energycane genotypes from within the state.*

Figure S1. Experiment Sites and Crops

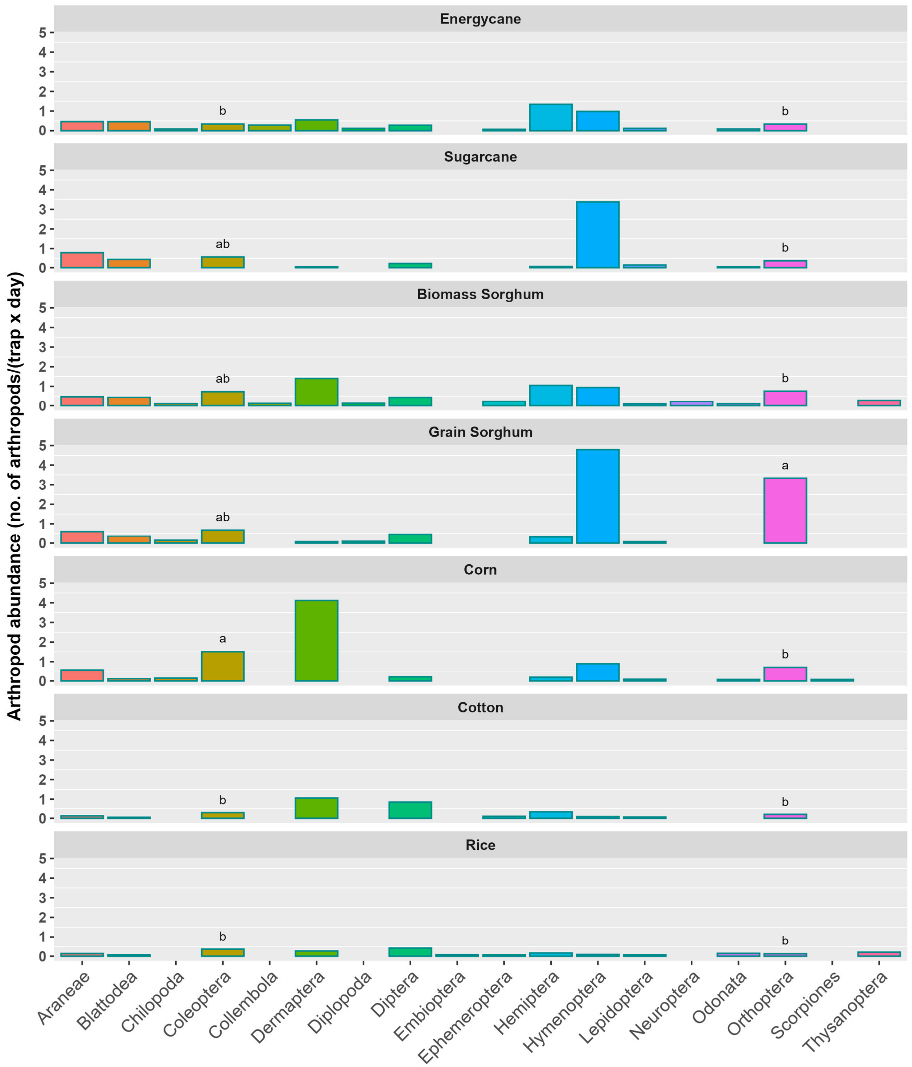

Figure S2. Distribution of arthropods among different taxonomic orders across different crops in three years (2020-2022). Different color bars represent different arthropod orders for easy visual differentiation.

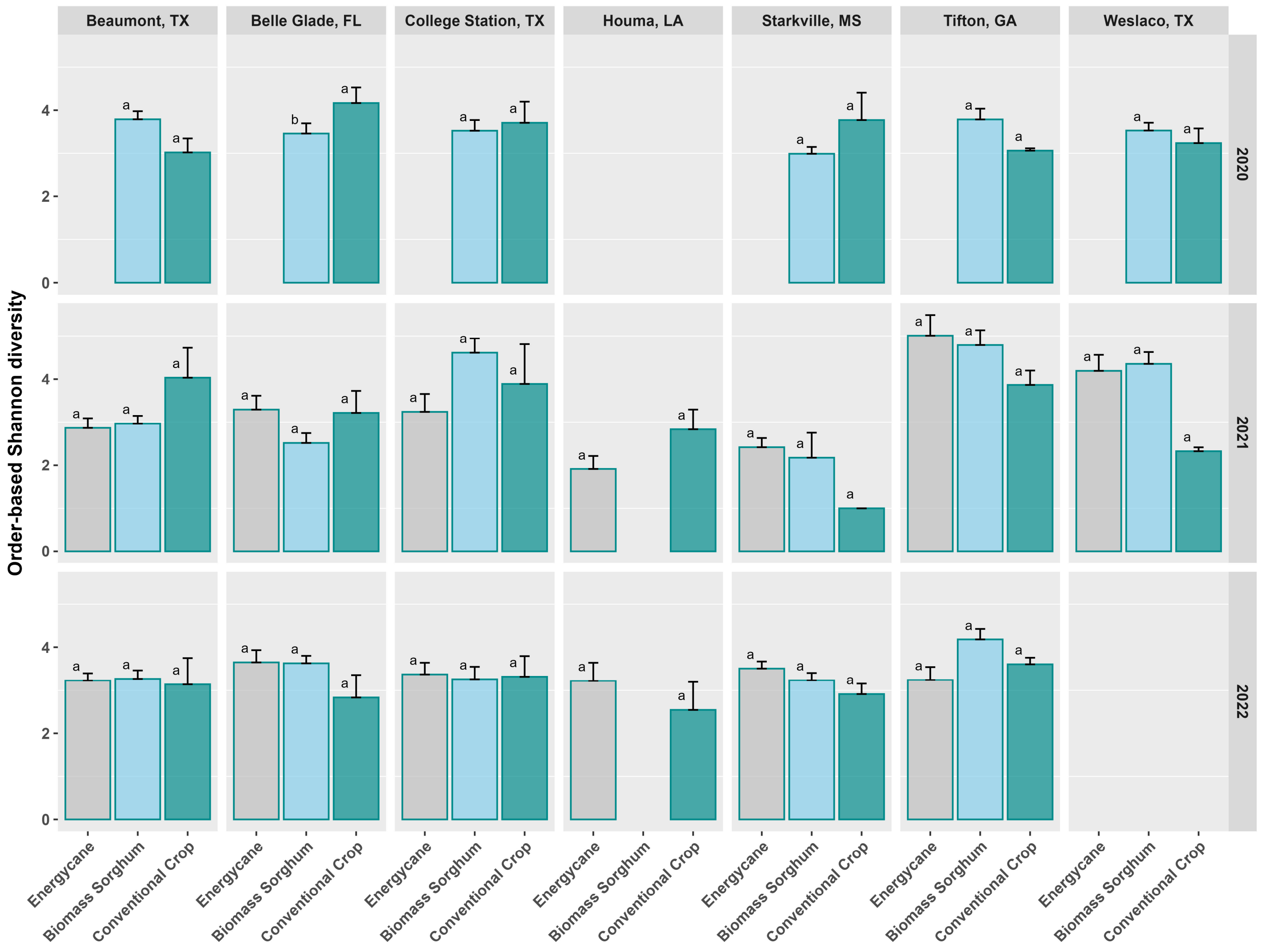

Figure S3. Order-based Shannon diversity from pitfall traps across seven sites and in three years (2020-2022) for three crop types. Crop types at a site and year having the same lowercase letter are not significantly different from each other at 0.05 with Tukey's HSD multiple comparison test. Error bars in the figure represent standard errors. Different color bars represent different crop types for easy visual differentiation.

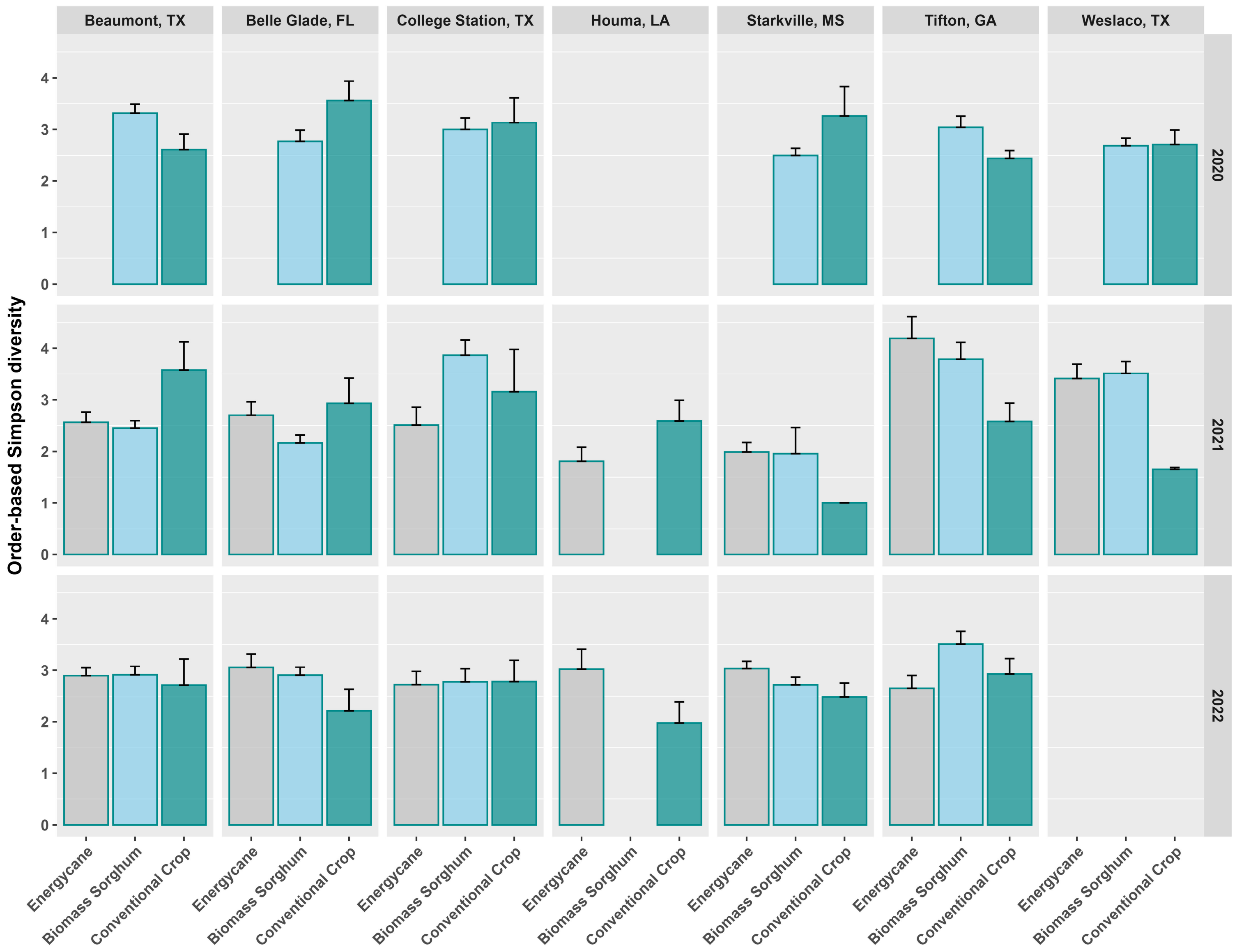

Figure S4. Order-based Simpson diversity from pitfall traps across seven sites and in three years (2020-2022) for three crop types. There were no significant differences among crop types at 0.05 with Tukey's HSD multiple comparison test. Error bars in the figure represent standard errors. Different color bars represent different crop types for easy visual differentiation.
